# Functional characterizations of rare *UBA1* variants in X-linked Spinal Muscular Atrophy

**DOI:** 10.12688/f1000research.11878.1

**Published:** 2017-09-04

**Authors:** Chris D. Balak, Jesse M. Hunter, Mary E. Ahearn, David Wiley, Gennaro D'urso, Lisa Baumbach-Reardon

**Affiliations:** 1 Translational Genomics Research Institute (TGen), Phoenix, Arizona, 85004, USA; 2 Clinical Genomics, Ambry Genetics, 15 Argonaut, Aliso Viejo, California , 92656, USA; 3 Department of Molecular and Cellular Pharmacology, Miller School of Medicine, University of Miami, Miami, Florida , 33101, USA; 4 Department of Medicine, Division of Clinical Data Analytics and Decision Support, University of Arizona, College of Medicine-Phoenix, Arizona , 85004, USA

**Keywords:** X-linked spinal muscular atrophy, SMAX2, UBA1, ubiquitination, ubiquitin proteasome system, disease mechanisms

## Abstract

**Background: **X-linked spinal muscular atrophy (XL-SMA) results from mutations in the Ubiquitin-Like Modifier Activating Enzyme 1 ( *UBA1*). Previously, four novel closely clustered mutations have been shown to cause this fatal infantile disorder affecting only males. These mutations, three missense and one synonymous, all lie within Exon15 of the *UBA1* gene, which contains the active adenylation domain (AAD).

**Methods: **In this study, our group characterized the three known missense variants *in vitro*. Using a novel Uba1 assay and other methods, we investigated Uba1 adenylation, thioester, and transthioesterification reactions *in vitro* to determine possible biochemical effects of the missense variants.

**Results:** Our data revealed that only one of the three XL-SMA missense variants impairs the Ubiquitin-adenylating ability of Uba1. Additionally, these missense variants retained Ubiquitin thioester bond formation and transthioesterification rates equal to that found in the wild type.

**Conclusions: **Our results demonstrate a surprising shift from the likelihood of these XL-SMA mutations playing a damaging role in Uba1’s enzymatic activity with Ubiquitin, to other roles such as altering *UBA1* mRNA splicing via the disruption of splicing factor binding sites, similar to a mechanism in traditional SMA, or disrupting binding to other important *in vivo* binding partners.  These findings help to narrow the search for the areas of possible dysfunction in the Ubiquitin-proteasome pathway that ultimately result in XL-SMA. Moreover, this investigation provides additional critical understanding of the mutations’ biochemical mechanisms, vital for the development of future effective diagnostic assays and therapeutics.

## Introduction

### XL-SMA and SMA

Spinal muscular atrophies (SMA) are a group of genetic disorders characterized by the degeneration of lower motor neurons, resulting in moderate to severe muscle wasting and weakness ( Cifuentes-Diaz *et al.*, 2002; Wee *et al.*, 2010). The majority of documented cases are the result of genetic deletion events in the Survival of Motor Neuron 1, Telomeric ( *SMN1*) gene, which plays a crucial role in snRNP biogenesis and is key in mRNA processing and metabolism. This results in *SMN1*s deficiency in cells and in particular motor neurons ( Cifuentes-Diaz *et al.*, 2002; Wee *et al.*, 2010; Woo *et al.*, 2017). In 2008, a second gene was discovered as the cause of a rare, X-linked infantile form of SMA (XL-SMA) only affecting males. This more severe form results from mutations in the Ubiquitin-Like Modifier Activating Enzyme 1 ( *UBA1*) gene, whose protein product is the pinnacle enzyme in the ubiquitin proteasome system (UPS). This X-linked form of SMA presents very similarly to the classical Type 1 SMA (Werdnig-Hoffmann disease) phenotype, including profound muscle weakness, hypotonia, muscle atrophy, anterior horn cell loss, evidence of denervation by electromyogram (EMG), as well as neurogenic atrophy by muscle biopsy. However, additional phenotypic features of XL-SMA can include congenital hypotonia, multiple congenital contractures (arthrogryposis) +/- bone fractures, myopathic facies, undescended testes and early mortality by the end of the infancy stage ( Ramser *et al.*, 2008).

### UPS, Ubiquitination and Uba1

In cells, the rapid disassembly and recycling of proteins is equally as important as their synthesis, and essential for maintaining cellular and protein homeostasis. Studies on global protein turnover rates have produced average protein half-lives of only 24–48 hours in mammals ( Cambridge *et al.*, 2011; Toyama *et al.*, 2013) and breakdowns in this homeostasis give rise to a broad range of disorders including a large subset of neurodegenerative disorders ( Hetz & Glimcher, 2011). The cell’s principle proteolytic mechanism responsible for this assembly and disassembly is the ubiquitin-proteasome system (UPS). The Uba1 enzyme initiates the UPS cascade by activating the small protein Ubiquitin which is used in large part as a molecular “death tag” for target proteins. Additionally, Uba1 has shown to be involved in other essential roles such as regulation of cell cycle progression ( Joo *et al.*, 2007) and neuron development and function ( Rinetti & Schweizer, 2010).

Ubiquitination, or the post-translational modification of attaching ubiquitin molecules to target proteins, is the ultimate goal for the ubiquitin-proteasome proteolytic pathway leading up to direct degradation by the 26s proteasome. It consists of three main steps; each with their own key enzymes, and has been described in detail in several studies ( Cohen-Kaplan *et al.*, 2016; Dohmen *et al.*, 1995; Glickman & Ciechanover, 2002). Uba1 sits at the pinnacle of the UPS ubiquitination cascade, initiating a series of complex and well-regulated steps that are common to all known processes involving Ubiquitin conjugation ( Haas & Rose, 1982; Haas *et al.*, 1982). The first step is the activation of ubiquitin by Uba1. Free Ubiquitin in the cell is adenylated by Uba1 in the active adenylation domain (AAD) at the expense of ATP. This forms a tightly bound ubiquitin adenylate consisting of a high-energy bond between the C-terminal carboxylate of the ubiquitin and AMP, which is then immediately attacked by the catalytically active Cys632 and transferred to the second catalytic cysteine domain (SCCD) forming a Uba1-Cys632 thioester bonded to Ubiquitin’s last amino acid Lys76. Ubiquitin activation is complete when a second ubiquitin is adenylated and loaded in the AAD forming the doubly-loaded, ternary Uba1 complex consisting of a Uba1~Ubiquitin thioester and primed Ubiquitin adenylate. The second step is the conjugation of Ubiquitin to an E2 enzyme. This takes place via a transthioesterification reaction between the catalytic cysteine of Uba1 and a catalytic cysteine on one of the 35 human E2 enzymes. The final step is the ligation of the now E2-bound Ubiquitin to its target protein. This is accomplished in conjunction with one of hundreds of human E3 ligase enzymes, which recognize their own set(s) of target proteins and complete ubiquitin ligation by generally catalyzing an isopeptide bond between a lysine of the target substrate and the C-terminal Gly76 of Ubiquitin. This E1-E2-E3 process is typically repeated until four Ubiquitin molecules are linked to the substrate via Lys48 residues, which is the signal used by the 26S proteasome to recognize and degrade the attached protein.

### Uba1 and its human variants

Uba1 is a highly conserved protein in all eukaryotes from both a sequence and functional aspect ( Schäfer *et al.*, 2014). Knockout of this gene is embryonic lethal in lower eukaryotic species ( Kulkarni & Smith, 2008) and is presumed likewise in humans. Furthermore, *UBA1* has a high intolerance to sequence variation. No homozygous or hemizygous loss-of-function (LoF) mutations exist in the genome Aggregation Database (gnomAD), the largest reference sequencing database containing over 123,000 exomes and 15,500 whole genomes highly enriched for healthy individuals. Missenses variants are equally rare in the general population, with *UBA1*’s 3000+ exonic bps only having three common coding variants with a global frequency of 1% or higher ( Lek *et al.*, 2016). *UBA1* has two transcripts that result in two known isoforms in humans, Uba1a (118kDa) and Uba1b (110kDa), with the shorter isoform only lacking the first 40 amino acids containing a nuclear localization signal. Further mention of *UBA1* in this manuscript will refer to the longest isoform including the NLS. Uba1 is made up of several domains including inactive and active adenylation domains (IAD, AAD), a Ubiquitin fold domain (UFD) as well as first and second catalytic cysteine half domains (FCCH, SCCH). An absolutely critical residue for Uba1’s adenylation activity is found in the ATP-Mg ^2+^ binding site of the AAD at position 576, and mutation to an unreactive alanine residue results in near null rates of adenylation ( Tokgöz *et al.*, 2006). The equally important cysteine residue at 632 forms the covalent thioester bond with ubiquitin after adenylation.

Four closely clustered mutations have been shown to cause infantile X-linked SMA (XL-SMA) ( Dlamini *et al.*, 2013; Dressman *et al.*, 2007; Ramser *et al.*, 2008). All four mutations, three missense and one synonymous, lie in exon 15 - the active adenylation domain (AAD) - of the *UBA1* gene. The three missense mutations, p.M539I, p.S547G and p.E557V all lie closely together in the AAD. A fourth recurrent synonymous mutation, Uba1a c.1731C>T p.N577N, also lies within this region. This c.1731C>T variant is thought to be a methylation site and driving the recurrence of the C to T transition. It is anticipated that these mutations do not completely eliminate Uba1 function as this would almost certainly result in early embryonic lethality. The crystal structure of human Uba1 has yet to be determined, however the well-conserved homolog *S. cerevisiae* Uba1 has been resolved. The functional domains can be inferred as specific functions of Uba1 and have been confirmed in these animal systems. Uba1’s strict conservation throughout eukaryotes, specifically mammals, provides evidence these domains and functions of Uba1 are consistent in *Homo sapiens.*

## Materials and methods

### Reagents

Human-recombinant (HR) Ubiquitin, HR Fluorescein-labeled Ubiquitin and all HR E2 enzymes were purchased from Boston Biochem. Restriction enzymes used for primary mutant colony selection were purchased from New England Biolabs or Life Technologies. Purification reagents inorganic pyrophosphate (PP _i_), adenosine monophosphate (AMP) and Ubiquitin-agarose utilized during Uba1 purification processes were purchased from Sigma-Aldrich. Assay reagents purine nucleoside phosphorylase (PNP), pyrophosphatase and 7-methylthioguanosine (MesG) were components of the EnzChek pyrophosphate assay kit derived from the coupled assay developed by Wilson and Aldrich. Additional assay components hydroxylamine, adenosine triphosphate (ATP) and iodoacetamide were purchased from Sigma-Aldrich.

### Uba1 wild type and mutant plasmid construction

Plasmids (pENTR-D-TOPO) from Invitrogen’s Gateway system containing the coding nuclear form of the *Homo Sapiens* wild type (WT) *UBA1* and XL-SMA missense mutant forms p.M539I and p.S547G were generously provided by Dr. Gennaro D’Urso from the University of Miami Miller School of Medicine. Wild type and mutant *UBA1* coding sequences were excised from pENTR-D-TOPO and cloned into vector pDEST17 with a His-Patch thioredoxin tag in frame. Two additional mutants, the third and final XL-SMA missense variant p.E557V and an adenylation-handicapped control mutant p.D576A ( Tokgöz *et al.*, 2006), were generated from the pDEST17 Uba1 WT plasmid clones via the site-directed mutagenesis method of Liu *et al* ( Liu & Naismith, 2008). Mutagenic forward and reverse primer sequences synthesized by Integrated DNA Technologies for the p.E557V mutant were 5’ GGTCCTGACACGGTGCGCATCTATGATGAC and 5’ GTCATCATAGATGCGCACCGTGTCAGGACC, respectively. For variant p.D576A, forward 5’ CCAATGCCCTGGCCAACGTGGATGCC and reverse 5’ GGCATCCACGTTGGCCAGGGCATTGG primers were used. After plating overnight, single colonies were selected for initial mutation screening by mutation-specific restriction enzymes overlapping mutagenesis sites. The pDEST17-Uba1aD576A mutant gained an additional *Hpa*I (GTT’AAC) restriction site while pDEST17-Uba1aE557V gained an additional *Fsp*AI (rTGC’GCAy) restriction site. These plasmids were then propagated in NEB5α *Escherichia coli* cells and purified using either Zymo Research or Qiagen plasmid prep kits. Lastly, all positive mutant colonies were confirmed by Sanger sequencing at Arizona State University Biodesign Institute.

### Uba1 Wild Type and Mutant Expression and Purification

Plasmids pDEST17-Uba1-WT, pDEST17-Uba1M539I, pDEST17-Uba1S547G, pDEST17-Uba1E557V and pDEST17-Uba1D576A were transformed into Rosetta 2(DE3) competent cells (Novagen). Single colonies were selected and grown in 750mL of LB containing both 100 mg/ml Ampicillin and Chloramphenicol at 37°C. At OD _600_≈ 0.8-0.9, expression of Uba1 protein was induced with 0.5mM isopropyl β-D-1-thiogalactopyranoside (IPTG) at 16°C for 12 hrs. All of the following was performed at 4°C or on ice unless otherwise noted. Following induction, cells were collected by centrifugation (17000g) for 10mins. Cells were lysed via sonication with ice-cold 50mM HEPES + 150mM NaCl (pH8.0) (Buffer A) plus 0.5mM DTT, 0.1% TX-100 and protease inhibitor cocktail and subsequently centrifuged at 17,000xg for 30 minutes. Lysates were then combined with Mg ^2+^ and ATP (Sigma) to final concentrations of 10mM and 2mM, respectively. These were immediately added to Ubiquitin-linked agarose bead (Sigma) columns at room temperature. Columns were then washed with 20 bed volumes of Buffer A. Elution of Uba1 enzyme was performed with three 10-minute column incubations containing 1mL Buffer A plus 2mM AMP and PP _i_. For Uba1D576A, affinity purification was unsuccessful as anticipated. Therefore, the inserted 6xHisTag was used in combination with Cobalt beads for purification in the same method as above, except 10mM and 200mM Imidazole were used for washes and elution respectively. All elutions were then combined and dialyzed three times at 4°C against 3 Liters of Buffer A plus 0.5mM DTT over a period of 18 hours. All phosphate buffers/reagents were avoided to eliminate chances of trace PP _i_ and Pi contamination in subsequent assays.

### Continuous coupled hydroxamate–MesG adenylation assays

Continuous adenylation activity of wild type and mutant forms of the Uba1 enzyme were measured using the coupled hydroxamate-MesG method reported by Wilson and Aldrich ( Wilson & Aldrich, 2010). The overall adenylation–acylation reaction of Uba1 generates one molecule of PPi at the expense of ATP per ubiquitin conjugated to the enzyme. Pyrophosphatase then hydrolyzes PPi into two inorganic phosphates, which are then coupled to phosphorolysis of 7-methylthioguanosine (MesG), catalyzed by the enzyme purine nucleoside PNP, and generating a chromophoric guanine derivative. Reactions totaling 150μl were set up in 96-well half-area UVStar plates (Greiner) containing 100nM WT or mutant Uba1 enzyme, 50 mM HEPES pH 8.0, 150mM hydroxylamine, 0.1U purine nucleoside phosphorylase, 0.15U pyrophosphatase, 200μM MESG and varying concentrations of Ubiquitin and MgATP. Uba1 concentrations were determined by either the Bradford BCA method or Pierce 660nm assay. Reactions were initiated at 37°C with either Ubiquitin or MgATP, at indicated concentrations. Cleavage of MesG into the chromophoric product, 7-methylguanine, was monitored at 360nm on a Synergy Max plate reader (BioTek). All buffers and reagents were phosphate and pyrophosphate free. Working stocks of hydroxylamine were prepared fresh daily by combining 4M hydroxylamine, 7M NaOH and dH _2_O on ice in a 2:1:1 ratio respectively. Hydroxylamine and NaOH stocks were stored at 4°C for at least 1 month, however freshly prepared stocks of these reagents were later made every 2 months due to the degradation of these reagents over time.

### Fluorescent thioester and transthioesterification gel shift assays

Thioester bond formation rates of WT and mutant Uba1 and Ubiquitin were measured by fluorescent (Fl) SDS-PAGE time-course assays containing fluorescein-labeled Ubiquitin. Reactions containing 50mM HEPES (pH8.0), 5mM ATP, 25mM MgCl _2_, and 300nM Uba1 were preheated to 37°C. Reactions were started by the addition of Fluorescein conjugated Ubiquitin (UbFl) to a final concentration of 3μM. At the indicated time points, 18.75μL aliquots were removed from master reaction and quenched in 6.25μl of 4X Laemmli loading buffer (Bio-Rad) containing 100mM β-mercaptoethanol (BME). It is important to note that disulfide bonds are reduced by BME but thiolester bonds are not. Reactions were heated to 95°C for 10 min and then run on Stain-Free Any-kD SDS-PAGE gels (Biorad) and imaged using a light engine (UVP) for 480nM excitation and 520nm emission filters on a UVP imaging system (UVP). Gels were then crosslinked by exposure to UV light for 2 minutes then washed 3X for 5 min. in deionized water and imaged using a UV Transilluminator with a SYBR Gold filter to measure total protein. Transthioesterification rates of Ub from Uba1 to wild type E2 enzyme *UBE2E1* ( *formerly UbcH6*) were measured in a similar assay. 50mM HEPES (pH8.0), 5mM ATP, 25mM MgCl _2_, 200nM Uba1, and 1.5μM UbcH6 were preheated to 37°C. Reactions were started by the addition of UbFl to a final concentration of 5μM. At the indicated time points, 14μL aliquots were removed from master reaction and quenched in 14μl of 2X Laemmli loading buffer (Bio-Rad) containing 50mM BME. Gels were imaged as above and bands were quantitated using ImageJ software (v. 1.48, ( Schneider *et al.*, 2012). Total pixel intensity was measured for each band and background levels were subtracted and lane loads were normalized to total Uba1 protein.

## Results

### Sequence analysis and 3D modeling of XL-SMA Uba1 reveals strict residue conservation and localization

**Figure 1.**
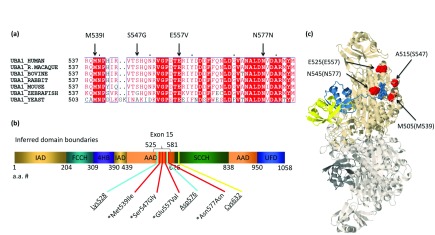
Uba1 conservation, domains, and modeling of XL-SMA mutations. ( **a**) XL-SMA variant residues show strict amino acid sequence conservation in nearly all eukaryotes from yeast to modern man. ( **b**) Schematic of the inferred domains of Uba1. Pathogenic variants and key residues are labeled. IAD = inactive adenylation domain, FCCH = first catalytic cysteine half domain, 4HB = 4 helix bundle, AAD = active adenylation domain, SCCH = second catalytic half domain. UFD = Ub fold domain. Known pathogenic variants marked with asterisk. Critical Adenylation and Thiolester residues are underlined. ( **c**) 3D protein modeling of *S. cerevisiae* Uba1 using Jmol modeling software. XL-SMA variants labeled in red, thiolestered Ub in yellow and non-covalently bound Ub-adenylate in blue. This figure has been reproduced and modified from Protein Data Bank public domain content (PDB ID: 4NNJ) under original permission from Schäfer *et al*. (2014). “Structure of the ubiquitin-activating enzyme loaded with two ubiquitin molecules.” Acta Crystallogr D Biol Crystallogr 70(Pt 5): 1311–1320.

To estimate the degree of sequence conservation in Uba1 and help elicit critical domain sequences surrounding the altered residues in patients with XL-SMA, the complete amino acid sequences of Uba1 enzymes were aligned and analyzed from a wide range of species using the Multalin program 5.4.1 ( Corpet, 1988). *Saccharomyces cerevisiae* (yeast), *Danio rerio* (zebrafish) and five well-studied mammalian Uba1 Multalin aligments are shown in Figure 1A. Despite the wide range of species aligned, the conservation was 100% identical in three of four variants. The fourth p.S547G variant diverges only at the level of yeast and zebrafish. This lends evidence of the requirement that amino acids spanning the ADD remain unchanged to maintain proper enzyme function, and it is important to note mammalian conservation was 100% among all species aligned in this analysis. A diagram of the various inferred domains of Uba1 and the clustering of XL-SMA mutations is show in Figure 1B.

The crystal structure for human Uba1 has yet to be determined, however in 2014 Schäfer *et al*. successfully resolved the crystal structure of Uba1 loaded with two Ubiquitin molecules and AMP (fully loaded) from *S. cerevisiae* with a resolution of 2.4Å. Using the 3D protein-modeling software Jmol ( Bowlin *et al.*, 2013), the three missense variants were highlighted to give spatial insights to possible effects of the variants ( Figure 1C). The p.M539I, p.S547G, p.E557V and p.N577 mutations (p.M505, p.A515, p.E525 and p.N545 in *S. cerevisiae,* respectively) all lie on the surface-exposed residues of the AAD.

### Synthesis and purification of Uba1 yielded active wild type, XL-SMA and adenylation mutants

**Figure 2.**
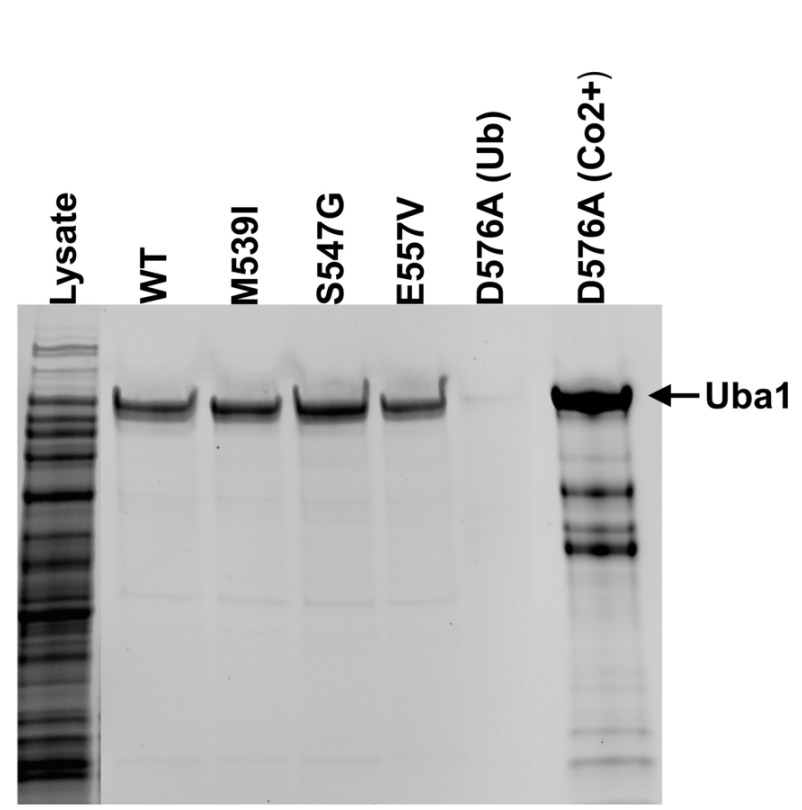
Expression and purification of Uba1. SDS-PAGE image of the purification of Uba1. Bands at ~118kDa indicate full length, active WT, p.M539I, p.S547G, p.E557V, Uba1 as purified by a thiolester-linkage capture column. As predicted, the p.D576A did not bind to the Ub-agarose. The p.D576A was purified by Co ^2+^ affinity column as seen in the far right lane.

Purification of solely full-length, catalytically active XL-SMA forms of Uba1 was necessary for reliable downstream experiments. We employed an affinity purification method first employed by Haas *et al*. for use in human erythrocytes, and modified it for use with Rosetta 2 (DE3) *Escherichia coli* competent cells for human protein over-expression. The method of purification relies solely on the catalytic ability of the Uba1 to perform adenylation and thioester bond formation with Ubiquitin. Therefore, highly purified, full-length and active Uba1 was obtained after stringent washing. Uba1 WT and XL-SMA variants were all successfully purified in this matter ( Figure 2). Yield was very similar and consistent for p.M539I, p.S547G, and WT Uba1 over numerous purifications. However, p.E557V Uba1 consistently resulted in a lower yield. Due to the Tokgoz *et al.* Uba1 p.D576A variant’s adenylation ability being reduced to virtually zero, this variant did not bind to the Ubiquitin-agarose column. Therefore, the 6xHisTag attached near the N-terminus of our Uba1 was used in combination with Cobalt Resin HisPur affinity beads (Thermo Fisher) for capture and subsequently eluted with 200mM imidazole.

### Uba1 *in-vitro* assay shows only p.E557V has reduced adenylation activity in XL-SMA mutants

**Figure 3.**
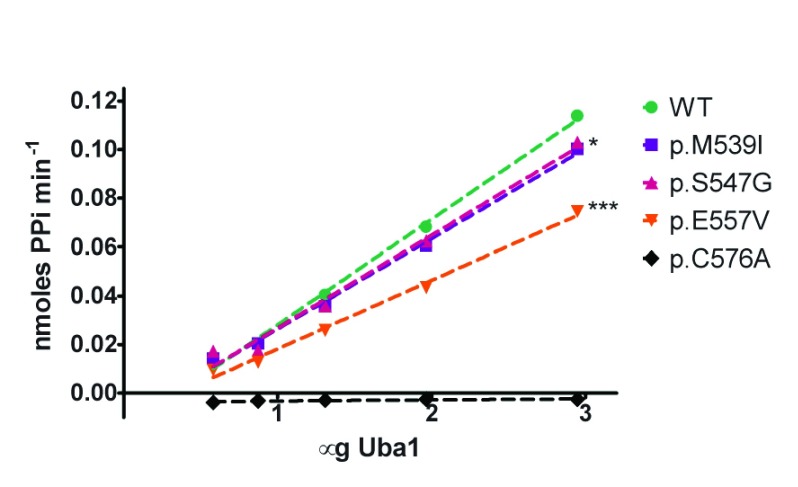
Uba1 adenylation activity plotted as a function of Uba1 enzyme amount. Kinetic assays were run in triplicate under saturating conditions (Mg ^2+^ (10mM), ATP (2mM), Ub (100µM)) at 37°C. Wild-type (WT) and XL-SMA variants were assayed with a range of Uba1 amounts in 50mM HEPES buffer pH 8.0. Linear regression of WT slope compared to each variant shows statistical significance for p.M539I (*P = 0.028) and p.E557V (***P<0.0001).

To test if XL-SMA missense variants alter the catalytic adenylation activity of Uba1, each missense variant was assayed *in vitro* against wild-type Uba1 using a novel kinetic assay adapted from Wilson *et al.* ( Wilson & Aldrich, 2010). Under saturating conditions of MgATP and Ubiquitin (2mM and 100μM, respectively) the adenylation activities of wild-type Uba1 and XL-SMA variants were assayed over a range of Uba1 concentrations, from 0–250nM. Adenylation-specific activity was measured by cleavage of ATP by Uba1, and the resulting PPi coupled to a colorimetric side reaction measuring at 360nm (see materials and methods). Missense variants p.M539I and p.S547G showed no statistically-reduced adenylation activity, despite their locations in the well-conserved AAD, while variant p.E557V showed a moderate decrease in activity ( Figure 3 and Table 1). The adenylation-crippling p.D576A variant was also generated in parallel with the XL-SMA variants, and included in the activity assay in order to serve as a negative control to validate the assay’s data. As predicted, variant p.D576A had essentially no detectable adenylation activity in the assay in relation to any of the other assays variants.

**Table 1.** Uba1 Adenylation Activity.

	specific activity (µmoles PPi min ^-1^ µg Uba1 ^-1^)	R square	P value
WT	0.04326 ± 0.001559	0.9961	
p.M539I	0.03705 ± 0.001490	0.9952	0.028
p.S547G	0.03807 ± 0.002588	0.9863	0.137
p.E557V	0.02817 ± 0.001372	0.9929	0.003
p.D576A	0.0004707 ± 0.0001916	0.6681	<0.0001

### Dependency of MgATP and Ubiquitin concentrations in Uba1 adenylation abilities

**Figure 4.**
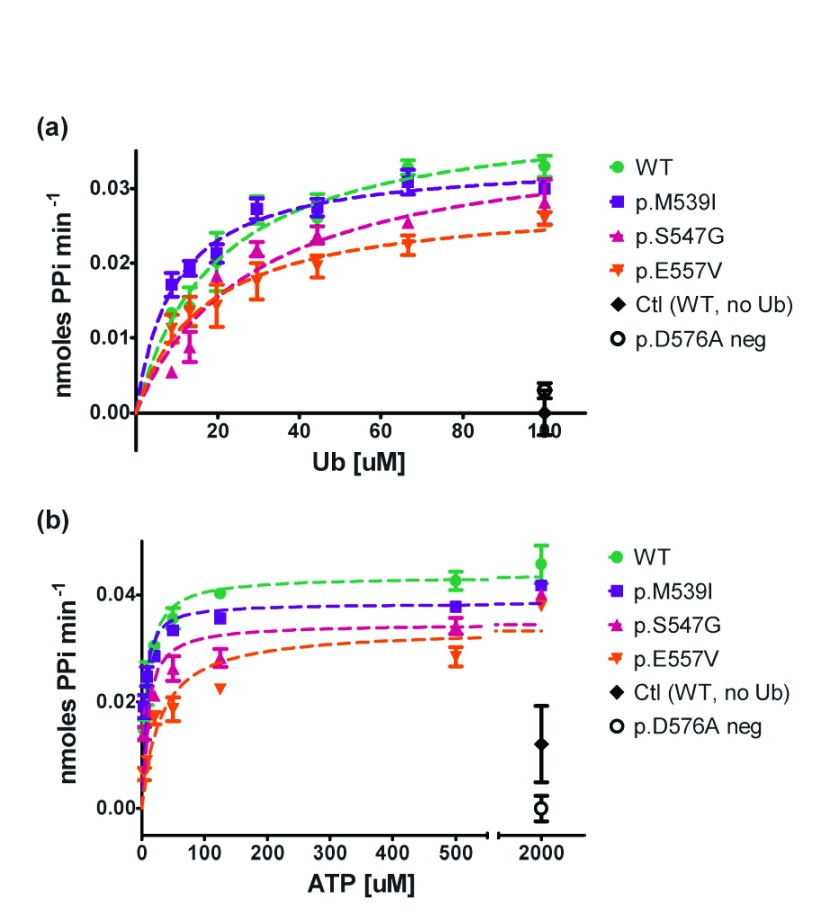
Uba1 adenylation activity as a function of Ub or ATP concentration. Kinetic assays (150µL) were run in duplicate or triplicate with 100nM WT Uba1 and XL-SMA variants in 50mM HEPES buffer at 37°C to determine initial linear rates from the first 10–20 minutes of the reaction. **A**) Michaelis-Menten graph of Uba1 adenylation activity as a function of Ub concentration with saturating ATP(2000nM). **B**) Michaelis-Menten graph of Uba1 adenylation activity as a function of ATP concentration with saturating Ub (100µM). Data was normalized to control reactions and graphed in GraphPad Prism. Km and Vmax values as well as 95% confidence intervals were also calculated in GraphPad Prism (see Table 2). Only p.E557V Km (ATP) did not have overlapping confidence intervals with WT values.

Despite the lack of significant changes in adenylation activity of the XL-SMA missense variants with respect to Uba1 concentrations for the p.M539I and p.S547G variants, further assays were carried out in an attempt to tease out any other significant differences under different assay conditions that might occur in the cell. To this end, varying concentrations of both ATP and Ubiquitin were assayed in separate experiments in triplicate ( Figure 4). Under saturating ATP concentrations (2mM), the concentration of Ub was varied between 100µM – 8.8µM. Michaelis–Menten kinetics did not reveal significant differences between the different Uba1 variants ( Figure 4 and Table 2). Similarly, under saturating Ub concentrations (100µM), ATP concentration was varied from 2mM down to 3.2nM. Only the p.E557V variant Uba1 had increased Km with 95% confidence intervals that did not overlap with WT Uba1, suggesting this variant has reduced affinity for ATP ( Figure 4 and Table 2).

**Table 2.** Uba1 Adenylation Activity Michaelis-Menten Values.

	Vmax (Ub)	95% CI	Km (Ub)	95% CI
WT	0.04063	0.03337 to 0.04789	19.89	9.507 to 30.27
p.M539I	0.03382	0.03117 to 0.03647	9.405	6.449 to 12.36
p.S547G	0.03763	0.02887 to 0.04639	28.72	12.12 to 45.32
p.E557V	0.0285	0.02362 to 0.03338	16.72	7.894 to 25.55
	Vmax (ATP)	95% CI	Km (ATP)	95% CI
WT	0.04346	0.04069 to 0.04623	7.491	4.670 to 10.31
p.M539I	0.0384	0.03619 to 0.04062	4.324	2.754 to 5.894
p.S547G	0.03461	0.03129 to 0.03793	8.547	3.724 to 13.37
p.E557V	0.03372	0.02964 to 0.03780	29.07	13.63 to 44.50

### Thioester bond formation in XL-SMA Uba1 variants

**Figure 5.**
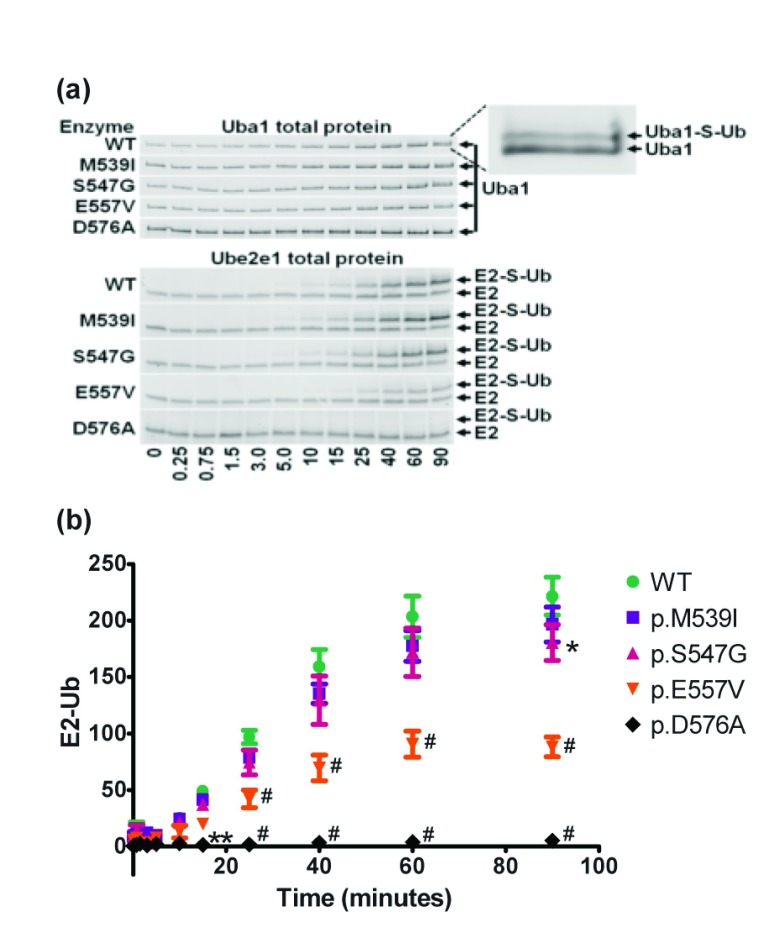
Uba1 transthioesteration of Ube2e1. WT and mutant forms of Uba1 were incubated with Ub and Ube2e1 in the presence of ATP and Mg2+ for the indicated time. Reactions were quenched with Laemli buffer, separated by SDS-PAGE and quantitated with ImageJ software. **A**) Gel images are representative of total protein images from three independent experiments. Inset for visualization of Uba1-S-Ub complex gel shift. Note the lack of E2-S-Ub formation in the p.D576A mutant control. **B**) Quantitation of the E2-S-Ub gel shift bands. Y-axis values are integrated Ube2e1-Ub band densities (in millions) normalized to total Uba1 band densities. Error bars represent the SEM. Symbols indicate statistical significance (#P<0.0001, **P<0.01, *P<0.05) compared to WT using 2-way ANOVA.

Numerous previous studies have shown the importance of Uba1’s Cys632, and its role in attacking the primed Ubiquitin-adenylate in the adenylation domain forming the thioester bond ( Haas & Rose, 1982; Haas *et al.*, 1982). With the catalytic adenylation activity of Uba1a showing no significant differences between wild type and XL-SMA mutants, the subsequent thioester bond formation reaction was investigated in order to determine if the mutations had any effect on the ability of Uba1’s catalytic cysteine to properly attack the Ubiquitin adenylate. To measure the covalent conjugation of Uba1’s Cys632 to Ubiquitin’s C-terminus Lys76 a fluorescently-labelled Ubiquitin time course assay was carried out ( Figure 5). Unfortunately, a time dependent, ATP-independent accumulation of fluorescence was found at the molecular weight of unmodified Uba1 in addition to the predicted ATP-dependent accumulation of fluorescence at a slightly higher-shifted molecular weight (data not shown). The interference from the ATP-independent accumulation of fluorescence made it impossible to determine Uba1-UbFl thiolester bond formation. We suspect the fluorescence accumulation without a shift in molecular weight was due to unreacted free fluorescein from the UbFl. While our thioester fluorescence assay’s data proved inconclusive, our purification of Uba1 active protein on Ub-agarose columns demonstrated clearly that all Uba1 enzymes tested, with the exception of the p.D576A mutant, readily form thiolester bonds. Interestingly, over many purification experiments, the yield of the p.E557V mutant Uba1 was consistently lower.

### Transthioesterification to E2 enzymes (UBE2E1) in XL-SMA Uba1 variants

In order to elicit any other type of enzymatic effect(s) the XL-SMA missense mutations might cause, we expanded the study into the final function performed by Uba1: the transfer of the activated Ubiquitin to a downstream E2 enzyme (transthioesterification).

After testing a kit of various E2 enzymes (Boston BioChem, data not shown), the Ubiquitin Conjugating Enzyme E2E 1 (UBE2E1, previously UBCH6) was chosen for an extended time course assay due to its well-studied function, properties, and adequate band separation between Ubiquitin dimers/trimers on SDS-PAGE. Again, with this assay, we found an ATP-independent accumulation of fluorescence at a molecular weight consistent with unmodified Ube2e1 (data not shown). We also found an ATP-dependent accumulation of fluorescent Ube2e1 consistent with Ubiquitin modification. Since the MW of Ube2e1-UbFl is significantly larger, we were able to accurately quantify the fluorescence of this gel shifted band ( Figure 5). Consistent with all of our previous findings, the p.M539I and p.S547G retained virtually all activity, though at the latest time point, p.S547G did reach statistical significance for a minute decrease in activity. The p.E557V rapidly reached a statistically-significant difference with less than half of wild-type activity.

## Discussion

X-linked spinal muscular atrophy is caused by rare disease-causing variants in *UBA1*. To date, one recurrent synonymous and three single-family missense mutations have been identified. These rare variants all reside in a 54 base-pair hotspot of the 3,174 nucleotide *UBA1* mRNA. To elucidate the biological dysfunction of Uba1 in XL-SMA, we performed novel *in vitro* biochemical assays on wild-type and pathogenic XL-SMA variants. 

The p.M539I, p.S547G and p.E557V missense XL-SMA variants all lie in the catalytic AAD domain of Uba1. It has been demonstrated the C-terminus tail of Ubiquitin must be brought together tightly with ATP in a narrow pocket, positioned in the correct orientation in order to be efficiently attacked by the catalytic cysteine in the adjacent SCCH domain ( Lee & Schindelin, 2008; Tokgöz *et al.*, 2006). To determine whether *UBA1* missense mutations affect this sensitive biochemical activity *in vitro*, we developed and performed a novel assay measuring Uba1 adenylation activity. The adenylation rates of Uba1 measured by our assay were somewhat slower in comparison to rates measured by others ( Tokgöz *et al.*, 2006; Wee *et al.*, 2000). However, our assay was designed to specifically isolate the adenylation activity of Uba1 in a kinetic assay by substituting Ub with a strong acceptor, hydroxylamine, thus separating the MgATP binding and adenylation half reaction away from binding of Ubiquitin and thioesterification half reaction. All other reported studies do not separate the half reactions and thus cannot accurately distinguish between altered adenylation and altered thioesterification. Using our novel assay, we measured the effect of alterations on the adenylation half reaction independently. Moreover, an endpoint that our rates strictly agree with others’ more sensitive assays was not an overriding goal in the novel assay, but to obtain catalytic rates between the XL-SMA variants relative to each other and wild type under the same conditions. Nevertheless, these differences noted above in assay conditions and reagents likely contributed to differences in rates and/or endpoints values between the current study and others.

Since all known XL-SMA missense variants lie in the AAD, we reasonably hypothesized these result in altered adenylation activity of Uba1. We expected a significant change, but not a complete elimination of enzymatic activity as complete loss of Uba1 function is inconsistent with life and cell survival at any level. To our surprise only 1 of 3 variants, p.E557V, had any significant reduction in enzymatic activity. It is a possibility the reduction is a result from the residue’s close proximity to the critical adenylating p.D576 residue which is why no significant change in activity was seen in the other missense variants located further upstream. Similarly, investigation of the transthioesterification from Uba1 to E2 enzymes, namely to Ube2e1, only p.E557V showed an apparent significant loss of transthioesterification signal compared to WT. This decrease in transthioesterification would be expected since the reduction in adenylation bottlenecks the reaction. However, in the presence of Ubiquitin, transthioesterification cannot be separated from adenylation and thioester half reactions, therefore additional dysfunction of the transthioesterification step cannot be ruled out. ****

In contrast to the p.E557V, our data suggest the p.M539I, and p.S547G missense variants in XL-SMA lead to negligible changes in relative enzymatic activity of Uba1 *in vitro*. These two variants showed no statistically significant effect, positive or negative, on Uba1’s adenylation activity of Ubiquitin *in vitro*. This discovery is somewhat in contrast with Tokgöz *et al*. (2006) previous findings of two adenylation-sensitive mutations in the ADD, specifically exon 15. Asp576 and Lys528 proved to be essential to the affinity of MgATP. Furthermore, substitutions to unreactive amino acids proved catalytically detrimental and effectively eliminating adenylation ability *in vitro*. However, given the complexity of protein domain folding and interaction currently one cannot accurately predict how each amino acid change could alter function. Vital for our studies, this virtually adenylation-dead p.D576A variant was generated and utilized as an optimal negative control for our assay’s own validation; and importantly showed no detectable activity levels in all assays.

As the p.M539I and the p.S547G mutations do not result in significant loss of *in vitro* catalytic activity, these observations suggest a different mechanism of pathology such as aberrant splicing, as is the suspected cause of disease associated with the c.1731C>T variant. Splice site and splice element blinding algorithms do suggest possible changes to splicing ( Lenski *et al.*, 2005). Another possibility is that p.M539I and the p.S547G mutations disrupt interactions with key binding partners. Allen *et al.* found that mutations in gigaxonin disrupt its interaction and binding with Uba1 ( Allen *et al.*, 2005). Gigaxonin ( *GAN*) is a class of BTB-Kelch proteins critical for specificity of the UPS. Mutations in *GAN* and several other BTB-Kelch proteins result in neuromuscular diseases with striking overlap with XL-SMA. This evidence demands that the effect of missense mutations on binding to gigaxonin and other BTB-kelch proteins be further explored.

The discovery of *UBA1* as the primary gene affected in XL-SMA was a major step forward in understanding the XL-SMA disease phenotype. Our results suggest a range of pathological effects of these rare variants in *UBA1*.

It is very interesting that the same conundrum is faced in our understanding the pathophysiology of autosomal recessive SMA, in which germ-line deletion of a highly important, ubiquitously expressed gene (SMN1), is also confined to targeted lower motor neuron destruction. It has been suggested that *UBA1* can rescue the *SMN1* phenotype in a murine model of autosomal recessive SMA ( Powis *et al.*, 2016). It will be of great interest in future investigations to better define the biological interactions of *SMN1* and *UBA1*; to see, if in fact, perturbations in SMN1 protein are also involved in pathogenesis of XL-SMA.

Dataset for Figure 2. Expression and purification of Uba1 Raw gel images used to create Figure 2 are labeled:
**“** Figure 2 **Uba1 purification total protein gel.TIF”**
Gel lanes are as follows
1.            Marker
2.            Clarified supernatant WT Uba1
3.            Clarified supernatant p.M539I Uba1
4.            Clarified supernatant p.S547G Uba1
5.            Clarified supernatant p.E557V Uba1
6.            Clarified supernatant p.D576A Uba1
7.            Empty
8.            Ub-agarose eluate WT Uba1
9.            Ub-agarose eluate p.M539I Uba1
10.          Ub-agarose eluate p.S547G Uba1
11.          Ub-agarose eluate p.E557V Uba1
12.          Ub-agarose eluate p.D576A Uba1
Figure 2 **Uba1 purification HIS tag total protein gel.TIF**
Gel lanes are as follows
1.            Marker
2.            Clarified supernatant p.D576A Uba1
3.            Co2+ flow through p.D576A Uba1
4.            Wash
5.            Empty
6.            Co2+ p.D576A Uba1 elution fraction 1
7.            Co2+ p.D576A Uba1 elution fraction 2
8.            Co2+ p.D576A Uba1 elution fraction 3
9.            Co2+ p.D576A Uba1 elution fraction 4
10.          Co2+ p.D576A Uba1 elution fraction 5
11          Co2+ p.D576A Uba1 elution fraction 6 Click here for additional data file. Copyright: © 2017 Balak CD et al. 2017 Data associated with the article are available under the terms of the Creative Commons Zero "No rights reserved" data waiver (CC0 1.0 Public domain dedication).

Dataset for Figure 3. Uba1 adenylation activity plotted as a function of Uba1 enzyme amount In order to create Figure 3, a pyrophosphate standard curve had to be generated first to determine the absorbance units/nanomoles of PPi (extinction coefficient). The raw data to generate this value can be found in “ Figure 3 data PPi std.csv”. The absorbance values at the 30 minute time point and the PPi concentration were used to generate the absorbance units/nanomoles of PPi value. This value was then used to determine the Uba1 adenlyation activity. Reactions were carried out as described in the materials and methods. “ Figure 3 data.csv” contains the raw data from this reaction set. The raw data were adjusted for the blank value at each time point, then converted to absorbance values to nanomoles of PPi using the value generated by the PPi standard curve. Linear portions of each reaction were selected and the slope determined. Data were converted to units shown in Figure 3. The data were then graphed and analyzed statistically using Graphpad Prism Version 5.04 software. Click here for additional data file. Copyright: © 2017 Balak CD et al. 2017 Data associated with the article are available under the terms of the Creative Commons Zero "No rights reserved" data waiver (CC0 1.0 Public domain dedication).

Data for Figure 4. Uba1 adenylation activity as a function of Ub or ATP concentration Figure 4a and Figure 4b were generated in much the same way as Figure 3 except varying concentrations of Ub or ATP were used in reactions with constant Uba1 enzyme concentrations. The raw data were adjusted for the blank value at each time point, then converted to absorbance values to nanomoles of PPi using the value generated by the PPi standard curve. Linear portions of each reaction were selected and the slope determined. Data were converted to units shown in Figure 3. The data were then graphed and analyzed statistically using Graphpad Prism Version 5.04 software. The raw absorbance values for Figure 4a are found in “ Figure 4a ub data.csv” and for Figure 4b are found in “ Figure 4b ATP data.csv”. Click here for additional data file. Copyright: © 2017 Balak CD et al. 2017 Data associated with the article are available under the terms of the Creative Commons Zero "No rights reserved" data waiver (CC0 1.0 Public domain dedication).

Data for Figure 5. Uba1 transthioesteration of Ube2e1 Figure 5a was generated from gel shift images from reactions described in materials and methods. Gel images are named “Figure_5_total_protein_r1_539.TIF”. Each gel image has a replicate number (e.g. “r1”) and a Uba1 protein identifier (e.g. “539” = p.M539I Uba1). Gel loads were loaded from left to right from reaction time points as indicated in Figure 5a. Figure 5b was generated by quantifying band intensity from the gel images using ImageJ software. The raw integrated band density values are found in file “Figure_5_band_intensities.csv”. The intensity of the nearby gel background was subtracted from the protein band intensities. The data were then graphed and analyzed statistically using Graphpad Prism Version 5.04 software. Click here for additional data file. Copyright: © 2017 Balak CD et al. 2017 Data associated with the article are available under the terms of the Creative Commons Zero "No rights reserved" data waiver (CC0 1.0 Public domain dedication).

## Data availability

The data referenced by this article are under copyright with the following copyright statement: Copyright: © 2017 Balak CD et al.

Data associated with the article are available under the terms of the Creative Commons Zero "No rights reserved" data waiver (CC0 1.0 Public domain dedication). 

F1000Research: Dataset 1. **Dataset for Figure 2. Expression and purification of Uba1**, 10.5256/f1000research.11878.d174721 ( Balak *et al.*, 2017a)

F1000Research: Dataset 2. **Dataset for Figure 3. Uba1 adenylation activity plotted as a function of Uba1 enzyme amount**, 10.5256/f1000research.11878.d174722 ( Balak *et al.*, 2017b)

F1000Research: Dataset 3. **Data for Figure 4. Uba1 adenylation activity as a function of Ub or ATP concentration**, 10.5256/f1000research.11878.d174723 ( Balak *et al.*, 2017c)

F1000Research: Dataset 4. **Data for Figure 5. Uba1 transthioesteration of Ube2e1**, 10.5256/f1000research.11878.d174724 ( Balak *et al.*, 2017d)
