# Supplementary material for: Functional characterizations of rare UBA1 variants in X-linked Spinal Muscular Atrophy
Source: F1000Res. 2017 Sep 4;6:1636. [Version 1] doi: 10.12688/f1000research.11878.1 (PMC5615770; doi:10.12688/f1000research.11878.1)
Supplement: Peer review history

# Peer review: Referee response for version 1

**DOI:** 10.5256/f1000research.12835.r25639

**Published:** 2017-09-25

**Authors:** Christine Beattie

**Affiliations:** 1 Department of Neuroscience, The Ohio State University, Columbus, OH, USA

This paper examines the functional properties of missense mutations in Ubiquitin-Like Modifier Activating Enzyme 1 (UBA1) found in patients with X-linked spinal muscular atrophy (XL-SMA). These rare mutations occur in the active adenylation domain of UBA1. To better understand how these mutations could be causing this motoneuron disease, the authors asked whether these mutations affected functional properties of this enzyme specifically UBA1 adenylation, thioester, and transthioesterification reactions *in vitro*.

 The data support that only the E557V mutation caused a significant decrease in UBA1 activity. The data were consistent from the different assays that analyzed UBA1’s ability to adenylate ubiquitin and transfer of the activated ubiquitin to a downstream E2 enzyme. However, this decrease was not complete and the other missense mutations did not show defects in UBA1 function in adenylation and downstream activities, thus the authors conclude that these functions of UBA1 do not contribute to the disease phenotype.

  

1. In at least two assays the E557V mutations had decreased enzymatic activity. Does this correlate with the severity of the disease? That is, do patients with the E557V mutations exhibit earlier disease onset or more severe disease phenotypes?
2. Figure 3. In the graph D576A is mis labeled as C576A
3. Figure 3: What statistical test was used for Fig. 3?
4. Figure 5: What posthoc analysis was used after the ANOVA?
5. Figure 4: It is not possible to see the UBA1-S-Ub complex gel shift in the upper gel. It would be beneficial to show them at higher magnification for at least one time point. 

I have read this submission. I believe that I have an appropriate level of expertise to confirm that it is of an acceptable scientific standard.

# Peer review: Referee response for version 1

**DOI:** 10.5256/f1000research.12835.r25643

**Published:** 2017-09-19

**Authors:** Barrington Burnett

**Affiliations:** 1 Department of Anatomy, Physiology and Genetics, Uniformed Services University of the Health Sciences (USUHS), Bethesda, MD, USA

Balak *et al *investigate the effect of one recurrent synonymous and three single-family missense mutations in the UBA1 gene, which this group and others have previously linked to X-linked spinal muscular atrophy. UBA1 is the first enzyme in the ubiquitin proteasome system which is responsible for tagging and degrading most cellular soluble proteins. Using *in vitro* assays, the authors investigated Uba1 adenylation, thioester, and transthioesterification reactions to determine possible biochemical effects of the missense variants. One hypothesis is that the disease causing UBA1 mutations result in loss of ubiquitin activation function. The authors present compelling data that ubiquitin activation and the transferred of the charged ubiquitin to an ubiquitin conjugating enzyme, E2, is not disrupted by the patient-derived mutations. Together the biochemical assays suggest that the mechanism of action may be alternative splicing of the UBA1 mutant gene product or selective ubiquitin transfer unto a yet to be determined E2 enzyme. Identifying the putative splice variant(s) or the aberrant E2 interaction and the downstream targets could shed light on why UBA1 mutations lead to X-linked SMA.

 This is a well written and elegantly done biochemical study. 

 Minor concern: It is unclear why the first letter in ubiquitin is capitalized throughout the manuscript.

I have read this submission. I believe that I have an appropriate level of expertise to confirm that it is of an acceptable scientific standard.
